# Supplementary material for: Frequency, complications, and mortality of inhalation injury in burn patients: A systematic review and meta-analysis protocol
Source: PLoS One. 2024 Apr 23;19(4):e0295318. doi: 10.1371/journal.pone.0295318 (PMC11037524; doi:10.1371/journal.pone.0295318)
Supplement: Attachment — Submitted filename: Review.docx [file pone.0295318.s002.docx]

I read with great interest the paper titled “Incidence and mortality in burn patients due to inhalation injury: a systematic review and meta-analysis protocol”.

The rational of this protocol is captivating. However, some changes are requested in order to improve the scientific quality (***ie***; writing style and methods) of this protocol.

Here are my remarks classified by themes

**GENERAL REMARKS**

*In order the shorten the Abstract and the paper, I recommend to abbreviate

inhalation injury as II and inhalation injuries as IIs at the first use

Invasive mechanical ventilation as IMV at the first use

*Change, when appropriate, over all the paper “mechanical ventilation” by “invasive mechanical ventilation”

*The paper need to be checked by a person fluent in English (or it is possible to use ChatGPT 3.5 to correct and improve the medical writing).

*Discordance between text and table 1: is it 19 years or 1 years?

*Change gender by sex. Please see the AMA recommendation related to the use of sex and gender: <https://www.ama-assn.org/press-center/press-releases/ama-adopts-new-policies-2018-interim-meeting>

*Delete the abbreviations’ list

**TITLE**

It does not really describe the aims of the study, since only the primary objective (Incidence and mortality) was reported. I recommend (if possible) to improve the title by including the other aims.

**ABSTRACT**

L71: Morbidity and mortality of what? More precision is needed.

L74-76: change “It is important to recognize early and know the factors associated with this type of commitment to better conduct and thereby improve its evolution” by “It is important to recognize II early and know the factors associated with it to better conduct and thereby improve its evolution”.

L88: change as ‘variables and the total number of participants”.

**KEYWORDS**

Please:

Opt for MeSH terms

Do not cite as keywords some terms previously used in the title or the abstract

Classify the key words in alphabetical order

**INTRODYCTION**

Major remark: this important section MUST be organised into three paragraphs (not 9 as done in this paper). The first paragraph should describe what is known. The second paragraph must detail what is unknown. The last paragraph should be reserved to your aims. Therefore, I kindly ask you to reorganise your introduction.

Major remark: Several sentences are lacking references (see below)

L111: add a reference after monitoring

L112: Morbidity and mortality of what?

L113: add a reference after damage

L114: can you add the exact date (month, year) where data (***ie***; 11,000,000 new cases and 180,000 deaths worldwide every year, and in Brazil, there are around 1,000,000 incidents annually) were collected? Moreover add at least 2 references to argue your data

L116: the expression “of these” is related to what worldwide data or Brazilian data?

L130: Change “II represents severity..”

L132-134: correct the English style, since the sentence is unclear “In addition…….condition [14]

L157: add a reference after life support

L160: add a reference after complications

L166-178: the subsection OBJECTIVES must reintegrate the Introduction section (must be the third paragraph). Here is a proposition.

The primary objective of this study will be to evaluate the incidence and mortality in burn patients due to IIs. The secondary objectives will be to evaluate the ***i)*** Association between IIs and the need for IMV in burn patients; ***ii)*** Association between IIs and respiratory complications in burn patients; ***iii)*** Association between IIs and clinical complications related to the various organ systems in burn patients; and ***iv)*** Factors associated with IIs in burn patients.

**METHODS**

MAJOR POINT: avoid redundancy between text and tables 1 and 2.

L185: delete (PROSPERO)

L225: delete over 20 years of age

L240: delete (RCTs)

L254: MeSH not Mesh

L255-256: change as “Entree terms (<https://www.embase.com>)”

L282: over all the paper use the following model when describing the reviewers: (JA and DS in the authors’ list).

L295: add a reference after “Newcastle Ottawa Scale Tool”.

L318: how you will contact the studies' authors ?

L325: delete (SMD)

**DISCUSSION**

L356: add a reference after severe condition

**REFERENCES**

Ref 1 to 4: please cite the English titles..

**TABLE 1.**

Population: 19 years not 17 years, sex not gender,

Outcomes: Secondary: need for, “mechanical ventilation” by “invasive mechanical ventilation”

Type of study: should you report that only RCT will be included (better that Observational studies). In fact, there is a discordance between text and table 1 regarding this point (see L240)

Type of study: do will exclude letters to editor and editorials
