# Supplementary material for: Frequency, complications, and mortality of inhalation injury in burn patients: A systematic review and meta-analysis protocol
Source: PLoS One. 2024 Apr 23;19(4):e0295318. doi: 10.1371/journal.pone.0295318 (PMC11037524; doi:10.1371/journal.pone.0295318)
Supplement: Attachment — Submitted filename: Authors response.docx [file pone.0295318.s003.docx]

Dear Editor,

Thank you for considering the paper "Frequency, complications and mortality of inhalation injury in burn patients: a systematic review and meta-analysis protocol" by Ávila et al., for publication in the PLOS One and allowing us to re-submit this revised manuscript.

The authors are grateful to the reviewers for the careful appraisal, positive comments, and helpful criticisms. Suggested changes have been addressed. We believe that the quality of the manuscript has been improved and hope that it now meets the quality required for publication in the International PLOS One.

A detailed point-by-point response is given below.

We are looking forward to your decision regarding the suitability of the revised version of this paper for the Journal.

Thank you very much in advance,

**Juliana Elvira Herdy Guerra Avila, MD, MSc***

**** Corresponding author***

E-mail: [julianabsb2010@hotmail.com](mailto:julianabsb2010@hotmail.com)

<https://orcid.org/0000-0003-2304-2046>

Programa de Pós-Graduação em Ciências da Saúde, Faculdade de Ciências de Saúde - Universidade de Brasília-UnB, Brasilia (DF), Brazil

**Editor Comments**

**#1 -** The manuscript presents a protocol for a systematic review and meta-analysis on the epidemiology and burden of inhalation burns, with a focus on their association with invasive mechanical ventilation and poor prognosis. The introduction section provides an overview of the research problem and its significance. It effectively introduces the topic of inhalation burns, emphasizing their morbidity and mortality impact worldwide. The authors contextualize the importance of this study by mentioning the global prevalence of burn cases and the substantial healthcare burden it presents. They also draw attention to the prevalence of inhalation injuries in burn patients, which is a good strategy to underscore the significance of this study.

**Response:** Thank you for the positive comment.

**#2 -** However, this paragraph is very long and the introduction could be enhanced by discussing specific gaps in the existing literature related to the frequency of inhalation burns and their association with invasive mechanical ventilation and complications. While the authors mention that there is room for advancement in the treatment of respiratory impairments following inhalation burns, a more explicit statement about the current knowledge limitations and the clinical relevance of addressing these gaps would strengthen the introduction.

**Response:** Thank you for the comment. We reorganized and rewrote the Introduction section, adding reference to existing gaps in the literature and the clinical relevance of addressing them. The changes in the Introduction are quoted below.

"Burns are tissue traumas caused by energy transfer (thermal, chemical, electrical, radiation) [1,2] and occur with variable local and systemic inflammatory responses according to the intensity, location, and the affected area depth [3]. Due to the severity of their conditions, most patients require treatment in specialized units with intensive support and monitoring [4]. The consequences of burns represent a public health problem, ranging from physical incapacity and psychological and social damage to death [4]. As per a World Health Organization Fact Sheet dated October 2023, there are over 11,000,000 cases worldwide annually, resulting in 180,000 deaths [5]. Only in Brazil, from 2015 to 2020, there were 19,772 deaths from burns, as delineated by data provided by the Brazilian Department of Health [6]. According to US statistics from the National Inpatient Sample and the National Burn Repository, 40,000 hospitalizations are estimated yearly due to burns in the United States, with about 5% presenting inhalation injuries (IIs) [6,7]. Approximately 33% of all burn patients will require invasive mechanical ventilation (IMV), which increases significantly with II [8].

The diagnosis of respiratory system involvement is essentially clinical and can be complemented by bronchoscopy and other radiological and laboratory tests [9]. In ideal conditions, bronchoscopy should be performed in the first 24 hours in all patients with a history of smoke inhalation and is considered the gold standard for this type of evaluation [10,11]. When present, IIs significantly impact patient outcomes, increasing fluid needs during resuscitation, pulmonary complications, and mortality [12-14], serving as a marker of severity and an independent risk factor for death [13,14], especially in patients with over 20% of body surface area burned [15,16]. Besides, contrary to the recent advancements in the treatment of cutaneous burn injuries, the complex treatment of the IIs remains a challenging frontier since the pathophysiology is not fully understood, the diagnostic criteria remain unclear, the interventions are often ineffective, and the mortality remains high [17-20].

The treatment of IIs is traditionally performed through respiratory support with 100% oxygen, hyperbaric oxygen therapy, and/or protective IMV [9,21,22]. However, questions regarding the best way to identify and classify respiratory tract involvement, whether all patients should be intubated and receive IMV, which IMV mode is best indicated, and issues related to systemic toxicity are essential points that must be better elucidated [21].

In this context, the primary purpose of this study will be to assess the evidence regarding the frequency and mortality of II in burn patients. The secondary purposes will be to assess the evidence regarding the association between IIs and respiratory complications (pneumonia, airway obstruction, acute respiratory failure, acute respiratory distress syndrome - ARDS), need for IMV and complications in other organ systems, and highlight factors associated with IIs in burn patients and prognostic factors associated with acute respiratory failure, need for IMV and mortality of II in burn patients."

**#3 -** The methods section is comprehensive and well-structured. It outlines the study design, research question, inclusion and exclusion criteria, and the data sources clearly. The use of the PRISMA guidelines for conducting the systematic review and meta-analysis is appropriate and enhances the rigor of the study. One notable strength is the registration of the protocol on PROSPERO, which adds transparency and ensures that the review is conducted following a predefined plan. The PICOS criteria are also well-defined, and the inclusion and exclusion criteria are clearly outlined. It's a robust framework for selecting studies.

**Response:** Thank you for the positive comment.

**#4 -** However, the methods section could benefit from additional details on the search strategy, such as the specific search terms used in the databases and the strategy for manual reference searching. These details would provide transparency and aid in replication.

**Response:** Thank you for the comment. A table showing the complete search strategy for all databases was included. We will use the backward and forward snowballing for manual reference searching. We also included worldwidescience.org in the grey literature search. We rewrote the Search Strategy in Methods to clarify this aspect.

The changes are quoted below.

"The complete search strategy for all databases is shown in Table 2".

| **Database** | **Search** |
| --- | --- |
| PUBMED/ MEDLINE | #1: "Burns, Inhalation" [MeSH] OR (Inhalation Burns) OR (Burn, Inhalation) OR (Inhalation Burn)  #2: "Smoke Inhalation Injury" [MeSH] OR (Inhalation Injury, Smoke) OR (Injury, Smoke Inhalation) OR (Inhalation Injuries, Smoke) OR (Injuries, Smoke Inhalation) OR (Smoke Inhalation Injuries)  #3: "Epidemiology" [MeSH] OR (Social Epidemiology) OR (Epidemiologies, Social) OR (Epidemiology, Social) OR (Social Epidemiologies)  #4: #1 OR #2 AND #3 |
| EMBASE | #1: "Lung burn" exp: explode all trees  #2: “Epidemiology”[MeSH]: explode all trees  #3: #1 AND #2 |
| LILACS / BVS | #1: mh:" Queimaduras por Inalação" OR (Burns, Inhalation) OR (Quemaduras por Inhalación) OR (Brûlures par inhalation) OR (mh:C26.200.322)  #2: mh:“Lesão por Inalação de Fumaça” OR (Smoke Inhalation Injury) OR (Lesión por Inhalación de Humo) OR (Lésion par inhalation de fumée) OR (mh: C26.200.322.800)  #3: mh:”Epidemiologia” OR (Epidemiology) OR (Epidemiología) OR (Épidémiologie) OR (Epidemiologia Social) OR (mh: H02.403.720.500) OR (mh: SP5.001) OR (mh: SP8.946.702.667.586)  4: #1 OR #2 AND #3 |
| SCOPUS | #1: "Burns, Inhalation" OR (Inhalation Burns) OR (Burn, Inhalation) OR (Inhalation Burn)  #2: "Smoke Inhalation Injury" OR (Inhalation Injury, Smoke) OR (Injury, Smoke Inhalation) OR (Inhalation Injuries, Smoke) OR (Injuries, Smoke Inhalation) OR (Smoke Inhalation Injuries)  #3: "Lung burn"  #4: "Epidemiology" OR (Social Epidemiology) OR (Epidemiologies, Social) OR (Epidemiology, Social) OR (Social Epidemiologies)  #5: #1 OR #2 OR #3 AND #4 |
| Web of Science | #1: "Burns, Inhalation": explode all trees  #2: "Smoke Inhalation Injury": explode all trees  #3: "Epidemiology": explode all trees  #4: #1 OR #2 AND #3 |
| CINAHL | #1: "Burns, Inhalation": explode all trees  #2: "Smoke Inhalation Injury": explode all trees  #3: "Epidemiology or incidence or prevalence or occurrence": explode all trees  #4: #1 OR #2 AND #3 |

In addition, grey literature reports will be sourced through simplified searches on Google Scholar and worldwidescience.org.

Finally, forward and backward reference searches will be performed to identify any other potential studies that might have been missed in the search process (backward and forward snowballing)."

**#6 -** Additionally, the methods do not explicitly mention a plan for assessing the quality of the included studies, which is a crucial aspect of systematic reviews and meta-analyses.

**Response:** Thank you for the comment. We included the GRADE for the assessment of the quality of the evidence.

The changes in Methods are quoted below.

"*Quality of the evidence*

For this evaluation, the criteria of the Grading of Recommendations Assessment, Development and Evaluation (GRADE) working group will be used [28]. GRADE assesses the quality of the evidence based on the assessment of five domains: risk of bias, imprecision, inconsistency, indirectness, and publication bias [28]. Two reviewers will independently evaluate the quality of evidence of the included studies (JA and DS in the authors' list). The third reviewer (AI in the authors' list) will resolve the disagreements."

**#5 -** The authors are invited to check for the definition of "incidence" and "prevalence". I would suggest here the term "frequency" to avoid any inconsistency.

**Response:** Thank you for the comment. We changed "incidence" and "prevalence" to "frequency" throughout the text to avoid any inconsistency.

**#6 -** The authors are gently suggested to identify a particular phenotype of inhalation burns that is associated with invasive mechanical ventilation and poor prognosis. They may plan to define and search for exploratory factors and explanatory factors associated to this phenotype.

**Response:** Thank you for the comment. In the secondary purposes, we included factors associated with acute respiratory failure, IMV, and mortality. Identifying these factors may contribute to defining the phenotype of inhalation burns associated with IMV and poor prognosis.

The changes are quoted below.

INTRODUCTION

"In this context, the primary purpose of this study will be to assess the evidence regarding the frequency and mortality of II in burn patients. The secondary purposes will be to assess the evidence regarding the association between IIs and respiratory complications (pneumonia, airway obstruction, acute respiratory failure, acute respiratory distress syndrome - ARDS), need for IMV and complications in other organ systems, and highlight factors associated with IIs in burn patients and prognostic factors associated with acute respiratory failure, need for IMV and mortality of II in burn patients."

METHODS

"Outcomes evaluated

The primary outcomes will be:

- Frequency;
- Mortality.

Secondary outcomes will be:

- Respiratory complications: pneumonia, airway obstruction, acute respiratory failure, and ARDS;
- Need for IMV;
- Complications in other organ systems;
- Factors associated with IIs, need for IMV, complications in other organ systems, and mortality".

DISCUSSION

"(…) In this aspect, especially the identification of factors associated with acute respiratory failure, need for IMV, and mortality may contribute to defining the phenotype of inhalation burns that is associated with poor prognosis and clinical approaches that may have led to better outcomes, which can contribute to stricter monitoring of these patients and the institution of earlier clinical therapeutic approaches to improve outcomes for these patients".

**#7 -** They may also plan for including recent studies using specific treatments such as inhaled anticoagulants to prevent invasive mechanical ventilation, complications and poor outcome. I suggest to include some projected results with the description of different characteristics projected to be studied.

**Response:** Thank you for the comment. We changed the inclusion criteria to include clinical trials and observational studies. This change will also make it possible to evaluate specific treatments, such as inhaled anticoagulants and N acetylcysteine, as factors to prevent IMV, complications, and poor outcomes.

The changes are quoted below.

METHODS

"Observational studies and clinical trials that evaluated the frequency and mortality of burn patients exposed to smoke inhalation will be included".

DISCUSSION

"It is already known that II is an independent risk factor for mortality in patients with small and moderate burns [13]. In this respect, the management of IIs is essential and can vary from the conservative approach to more elaborate options involving drugs [23]. Specific treatments have been tested to prevent IMV, complications, and poor outcomes. Some studies observed that N acetylcysteine and inhaled anticoagulants (such as heparin) may effectively treat inhalation injury, significantly improving lung compliance and airway obstruction, reducing reintubation rates, increasing the number of ventilator-free days, and decreasing hospital length of stay, and mortality [40-43].

"(…) In this aspect, especially the identification of factors associated with acute respiratory failure, need for IMV, and mortality may contribute to defining the phenotype of inhalation burns that is associated with poor prognosis and clinical approaches that may have led to better outcomes, which can contribute to stricter monitoring of these patients and the institution of earlier clinical therapeutic approaches to improve outcomes for these patients".

**#8 -** The discussion section appropriately emphasizes the significance of inhalation injuries in burn patients and the challenges associated with diagnosis and treatment. It also highlights the need for further research in this area. However, it would be beneficial to discuss the potential implications of the projected study results in more detail. For example, how might the findings impact clinical practice, patient outcomes, or future research directions.

**Response:** Thank you for the comment. We reorganized and rewrote the Discussion. Especially in the last paragraph, we summarized the potential implications of the projected study results, as quoted below.

"Respiratory impairment is still a major challenge in clinical practice and a promising area for research, needing further studies to understand and act on this potentially severe condition [44]. In this systematic review, we aim to clarify the principal voids in the existing literature regarding fire-related II to guide future studies. Furthermore, the findings can contribute to diagnostic and management protocols for II in burned patients, which may improve health care and the prognosis of these people. In this aspect, especially the identification of factors associated with acute respiratory failure, need for IMV, and mortality may contribute to defining the phenotype of inhalation burns that is associated with poor prognosis and clinical approaches that may have led to better outcomes, which can contribute to stricter monitoring of these patients and the institution of earlier clinical therapeutic approaches to improve outcomes for these patients."

**Reviewer #1**

In this manuscript entitled "Incidence and mortality in burn patients due to inhalation injury: a systematic review and meta-analysis protocol" Id: PONE-D-23-20903, the authors present the protocol of a systematic review and meta-analysis focused on a hyper-specialized subject where this type of writing is lacking: Inhalation injury in critical burns patients. It is an interesting idea and a solid design and a clear and detailed methodology that is robust and a priori conforms to the norms for carrying out this type of study.

**Response:** Thank you very much for the positive comments.

Nevertheless, I raised some concerns and questions:
**#1 -** Note of form: I consider the introduction is very long (too much epidemiology))), many parts can be switched to the discussion (such as pathophysiology of the lung injury inhalation, diagnostic tools, therapeutic modalities, etc.)

**Response:** Thank you for the comment. We reorganized and rewrote the Introduction, adding reference to existing gaps in the literature and the clinical relevance of addressing them. Besides, some sentences of the Introduction were moved to the Discussion.

The changes are quoted below.

INTRODUCTION

"Burns are tissue traumas caused by energy transfer (thermal, chemical, electrical, radiation) [1,2] and occur with variable local and systemic inflammatory responses according to the intensity, location, and the affected area depth [3]. Due to the severity of their conditions, most patients require treatment in specialized units with intensive support and monitoring [4]. The consequences of burns represent a public health problem, ranging from physical incapacity and psychological and social damage to death [4]. As per a World Health Organization Fact Sheet dated October 2023, there are over 11,000,000 cases worldwide annually, resulting in 180,000 deaths [5]. Only in Brazil, from 2015 to 2020, there were 19,772 deaths from burns, as delineated by data provided by the Brazilian Department of Health [6]. According to US statistics from the National Inpatient Sample and the National Burn Repository, 40,000 hospitalizations are estimated yearly due to burns in the United States, with about 5% presenting inhalation injuries (IIs) [6,7]. Approximately 33% of all burn patients will require invasive mechanical ventilation (IMV), which increases significantly with II [8].

The diagnosis of respiratory system involvement is essentially clinical and can be complemented by bronchoscopy and other radiological and laboratory tests [9]. In ideal conditions, bronchoscopy should be performed in the first 24 hours in all patients with a history of smoke inhalation and is considered the gold standard for this type of evaluation [10,11]. When present, IIs significantly impact patient outcomes, increasing fluid needs during resuscitation, pulmonary complications, and mortality [12-14], serving as a marker of severity and an independent risk factor for death [13,14], especially in patients with over 20% of body surface area burned [15,16]. Besides, contrary to the recent advancements in the treatment of cutaneous burn injuries, the complex treatment of the IIs remains a challenging frontier since the pathophysiology is not fully understood, the diagnostic criteria remain unclear, the interventions are often ineffective, and the mortality remains high [17-20].

The treatment of IIs is traditionally performed through respiratory support with 100% oxygen, hyperbaric oxygen therapy, and/or protective IMV [9,21,22]. However, questions regarding the best way to identify and classify respiratory tract involvement, whether all patients should be intubated and receive IMV, which IMV mode is best indicated, and issues related to systemic toxicity are essential points that must be better elucidated [21].

In this context, the primary purpose of this study will be to assess the evidence regarding the frequency and mortality of II in burn patients. The secondary purposes will be to assess the evidence regarding the association between IIs and respiratory complications (pneumonia, airway obstruction, acute respiratory failure, acute respiratory distress syndrome - ARDS), need for IMV and complications in other organ systems, and highlight factors associated with IIs in burn patients and prognostic factors associated with acute respiratory failure, need for IMV and mortality of II in burn patients."

DISCUSSION

"Inhalation injury is a frequent condition following burn injury, notably increasing the frequency with the rise of the burn injury size and patient age [29,30]. Although there is already extensive experience managing IIs in burn patients, they still represent a great challenge, mainly due to their complex pathophysiological process that has not yet been fully clarified, where the involvement of several inflammatory cells, mediators, and cytokines has been demonstrated [19]. Diagnosis and accurate measurement of its damage are also complex, and therapies are essentially based on supportive measures [17].

In the IIs, the magnitude and location of the injury vary considerably according to the environment and the host factors [31]. In this respect, several factors should be considered, such as ignition source, concentration and solubility of inhaled substances, diameter and size of the particles in the smoke, exposure duration, temperature, and patient immune response [20,31,32]. Individuals aged 65 and beyond exhibit a mortality rate from burns exceeding the average six factors [33]. Due to diminished physiological reserves and comorbidities, managing this demographic poses a distinctive and formidable challenge. Multiple preexisting risk factors are manifest in older adults, encompassing an elevated susceptibility to infections, pulmonary diseases, and comorbidities [34]."

**#2 -** Regarding the primary objective: the authors announce throughout the manuscript to evaluate mortality and epidemiology. For the last, sometimes it is announced incidence and sometimes prevalence. Will it depend on the type of results collected from the different studies included? Or is it just a simple proportion of lung injury by inhalation among the total number of burns counted?
**Response:** Thank you for the comment. We changed "incidence" and "prevalence" to "frequency" throughout the text to avoid any inconsistency.

**#3 -** Page 8, L 194: I (exposure) = inhalation of toxic substances. What kind of toxic substances is covered here: Chemical irritants, toxic metals, asphyxiants, products of fires and combustion, other? All of them? Since lung damage differs depending on the inhaled substance...

**Response:** Thank you for the comment. We changed "toxic substances" to "Inhalation injury" to avoid any inconsistency. The change is quoted below.

"II associated with the burn event"

Table 1 - Exposure type – "Inhalation injury"

**#4 -** As exclusion criterion, the authors selected Studies that evaluated specific interventions in patients' victims of burn exposed to inhalation of gases and toxic substances. It would be preferable if they provide more detail about this type of specific intervention and the reason why they exclude studies that are interested in it.

**Response:** Thank you for the comment. We changed the inclusion and exclusion criteria to include clinical trials and observational studies. This will also make it possible to evaluate specific interventions as factors to prevent IMV, complications, and poor outcomes.

The changes are quoted below.

INCLUSION CRITERIA

"Observational studies and clinical trials that evaluated the frequency and mortality of burn patients exposed to smoke Inhalation".

EXCLUSION CRITERIA

"Studies in patients under 19 years of age will be excluded.

Studies presenting incomplete data, reviews, case series, case reports, and editorials will be excluded. Letters to the editor that do not report results from original data will also be excluded".

**#5 -** Relating to the 4 secondary objectives which evaluate the factors of association between: 1-inhalation injuries / need for invasive ventilation, 2- inhalation injuries / respiratory complications, 3-inhalation injuries / clinical complications and 4-factors associated with inhalation injuries: For the first: the attributability of the need for mechanical ventilation to the inhalation injury is not guaranteed (indeed the ARDS induced by skin burns is in itself a frequent cause of need to ventilation). Thus, the causal link the effect is far from being established in the various studies to be collected. Do the authors plan to adjust the selection criteria to minimize this bias? For the 4 association studies, are there predefined factors (according to the literature) that will be introduced in the analysis of each type of association?

**Response:** Thank you for the comment. Burn injury size and patient age are the most frequent factors associated with these outcomes cited in the literature. To minimize the risk of bias due to ARDS-induced and other respiratory complications that may be induced by skin burns without IIs and it is a frequent cause of need for IMV, we will only include studies that compared burn patients with and without II to evaluate the association between IIs and respiratory complications (pneumonia, airway obstruction, acute respiratory failure, acute respiratory distress syndrome), and need for IMV and complications in other organ systems, and the factors associated with IIs in burn patients. We also included the possibility of exploring subgroups based on the severity of confounding factors in the statistical analysis and emphasized this aspect in the Discussion.

The changes are quoted below, as quoted below.

METHODS

"Summary of results and statistical analysis

(…) For further comparisons concerning the extent of burn injury size, shock, or presence of infections, subgroup analysis will be performed if feasible.".

DISCUSSION

"Although most patients exposed to smoke inhalation evolve well, the development of respiratory injury significantly worsens the outcome of these patients with a significant increase in mortality and complications, including long-term sequelae [17,34-36]. Indeed, pulmonary complications following burns and II cause or directly contribute to 77% deaths [37,38]. Among the pulmonary complications, ARDS may develop early or several days after the exposure [39]. Although ARDS may also occur in burns without II, the clinical symptoms tend to worsen following IIs. In II, ARDS usually starts earlier, progresses with greater severity, and requires IMV for longer [13]. Furthermore, sepsis and acute respiratory failure are frequent causes of morbidity and mortality in patients with exclusive thermal burns, which may be even more prevalent in patients with IIs [13]."

**#6 -** Thank you for giving me the opportunity to review this paper
Cordially

**Response:** Thank you very much for carefully analyzing the study that strengthened the manuscript.

**Reviewer #2**

Smoke inhalation in burn victims is associated with morbidity and mortality. A review of the literature on the incidence and complications secondary to pulmonary burns would be of great help in setting up specific therapies in pulmonary damage.
The protocol of this study is well-structured and meets the recommendations of a research study.
**Response:** Thank you very much for the positive comments.

Comments
**#1 -** L 166 : objectives
178 Evaluate the factors associated with inhalation injuries in burn patients.
Replace this suggestion by "highlight prognostic factors associated with inhalation injuries in burn patients"

**Response:** Thank you very much for carefully analyzing the study that strengthened the manuscript. We changed the objectives as quoted below.

"In this context, the primary purpose of this study will be to assess the evidence regarding the frequency and mortality of II in burn patients. The secondary purposes will be to assess the evidence regarding the association between IIs and respiratory complications (pneumonia, airway obstruction, acute respiratory failure, acute respiratory distress syndrome - ARDS), need for IMV and complications in other organ systems, and highlight factors associated with IIs in burn patients and prognostic factors associated with acute respiratory failure, need for IMV and mortality of II in burn patients."

**Reviewer #3**

I read with great interest the paper titled "Incidence and mortality in burn patients due to inhalation injury: a systematic review and meta-analysis protocol".
The rational of this protocol is captivating.
**Response:** Thank you for the positive comment.

However, some changes are requested in order to improve the scientific quality (ie; writing style and methods) of this protocol.
Here are my remarks classified by themes

GENERAL REMARKS

**#1 -** *In order the shorten the Abstract and the paper, I recommend to abbreviate
inhalation injury as II and inhalation injuries as IIs at the first use
Invasive mechanical ventilation as IMV at the first use
*Change, when appropriate, over all the paper "mechanical ventilation" by "invasive mechanical ventilation"

**Response:** Thank you for the comment. We changed "mechanical ventilation" to "invasive mechanical ventilation" and used abbreviations throughout the manuscript as suggested.

**#2 -** *The paper need to be checked by a person fluent in English (or it is possible to use ChatGPT 3.5 to correct and improve the medical writing).

**Response:** Thank you for the observation. The English style was revised throughout the manuscript.

**#3 -** *Discordance between text and table 1: is it 19 years or 1 years?
**Response:** Thank you for the comment. In Table 1 – Exclusion Criteria, we changed "under 17 years of age" to "under 19 years of age", as quoted below.

"Patients under 19 years of age victims of burn (…)".

**#4 -** *Change gender by sex. Please see the AMA recommendation related to the use of sex and gender: https://www.ama-assn.org/press-center/press-releases/ama-adopts-new-policies-2018-interim-meeting
**Response:** Thank you for the comment. We changed the text in Table1, as quoted below.

"Adult patients of both sexes victims of acute burn regardless of magnitude or cause".

**#5 -** *Delete the abbreviations' list
**Response:** Thank you for the comment. We deleted the abbreviations' list.

TITLE
**#6 -** It does not really describe the aims of the study, since only the primary objective (Incidence and mortality) was reported. I recommend (if possible) to improve the title by including the other aims.

**Response:** Thank you for the comment. The title was modified as suggested, including the term "complications," which encompasses the study's secondary purposes, as it refers not only to clinical conditions but also to the severity of conditions associated with inhalation and the need for IMV".

The changes in the Title are quoted below.

"Frequency, complications, and mortality of inhalation injury in burn patients: a systematic review and meta-analysis protocol "

ABSTRACT
**#7 -** L71: Morbidity and mortality of what? More precision is needed.
**Response:** Thank you for the comment. We changed the sentence, as quoted below.

**"**Burns are tissue trauma caused by energy transfer and occur with a variable inflammatory response. The consequences of burns represent a public health problem worldwide.

**#8 -** L74-76: change "It is important to recognize early and know the factors associated with this type of commitment to better conduct and thereby improve its evolution" by "It is important to recognize II early and know the factors associated with it to better conduct and thereby improve its evolution".
**Response:** Thank you for the comment. We excluded this sentence in the revised manuscript.

**#9 -** L88: change as 'variables and the total number of participants".

**Response:** Thank you for the comment. We changed the sentence as suggested. The change is quoted below.

"(…) Data will be synthesized through continuous (mean and standard deviation) and dichotomous (relative risk) variables and the total number of participants. (…)".

KEYWORDS
**#10 -** Please: Opt for MeSH terms
Do not cite as keywords some terms previously used in the title or the abstract
Classify the key words in alphabetical order

**Response:** Thank you for the comment. We changed the keywords, as quoted below.

“Burns, Inhalation; Inhalation exposure; Prognosis; Respiration, Artificial; Smoke inhalation injury”

INTRODUCTION
**#11 -** Major remark: this important section MUST be organized into three paragraphs (not 9 as done in this paper). The first paragraph should describe what is known. The second paragraph must detail what is unknown. The last paragraph should be reserved to your aims. Therefore, I kindly ask you to reorganize your introduction.

**Response:** Thank you for the comment. We reorganized and rewrote the Introduction as quoted below.

"Burns are tissue traumas caused by energy transfer (thermal, chemical, electrical, radiation) [1,2] and occur with variable local and systemic inflammatory responses according to the intensity, location, and the affected area depth [3]. Due to the severity of their conditions, most patients require treatment in specialized units with intensive support and monitoring [4]. The consequences of burns represent a public health problem, ranging from physical incapacity and psychological and social damage to death [4]. As per a World Health Organization Fact Sheet dated October 2023, there are over 11,000,000 cases worldwide annually, resulting in 180,000 deaths [5]. Only in Brazil, from 2015 to 2020, there were 19,772 deaths from burns, as delineated by data provided by the Brazilian Department of Health [6]. According to US statistics from the National Inpatient Sample and the National Burn Repository, 40,000 hospitalizations are estimated yearly due to burns in the United States, with about 5% presenting inhalation injuries (IIs) [6,7]. Approximately 33% of all burn patients will require invasive mechanical ventilation (IMV), which increases significantly with II [8].

The diagnosis of respiratory system involvement is essentially clinical and can be complemented by bronchoscopy and other radiological and laboratory tests [9]. In ideal conditions, bronchoscopy should be performed in the first 24 hours in all patients with a history of smoke inhalation and is considered the gold standard for this type of evaluation [10,11]. When present, IIs significantly impact patient outcomes, increasing fluid needs during resuscitation, pulmonary complications, and mortality [12-14], serving as a marker of severity and an independent risk factor for death [13,14], especially in patients with over 20% of body surface area burned [15,16]. Besides, contrary to the recent advancements in the treatment of cutaneous burn injuries, the complex treatment of the IIs remains a challenging frontier since the pathophysiology is not fully understood, the diagnostic criteria remain unclear, the interventions are often ineffective, and the mortality remains high [17-20].

The treatment of IIs is traditionally performed through respiratory support with 100% oxygen, hyperbaric oxygen therapy, and/or protective IMV [9,21,22]. However, questions regarding the best way to identify and classify respiratory tract involvement, whether all patients should be intubated and receive IMV, which IMV mode is best indicated, and issues related to systemic toxicity are essential points that must be better elucidated [21].

In this context, the primary purpose of this study will be to assess the evidence regarding the frequency and mortality of II in burn patients. The secondary purposes will be to assess the evidence regarding the association between IIs and respiratory complications (pneumonia, airway obstruction, acute respiratory failure, acute respiratory distress syndrome - ARDS), need for IMV and complications in other organ systems, and highlight factors associated with IIs in burn patients and prognostic factors associated with acute respiratory failure, need for IMV and mortality of II in burn patients."

**#12 -** Major remark: Several sentences are lacking references (see below)
L111: add a reference after monitoring

**Response:** Thank you for the comment. We rewrote the sentence, adding reference, as quoted below.

"(…) Due to the severity of their conditions, most patients require treatment in specialized units with intensive support and monitoring [4].] (…)".

**#13 -** L112: Morbidity and mortality of what?

**Response:** Thank you for the comment. We rewrote the sentence, as quoted below.

"(…) The consequences of burns represent a public health problem, ranging from physical incapacity and psychological and social damage to death [4] (…)"

**#14 -** L113: add a reference after damage

"(…) The consequences of burns represent a public health problem, ranging from physical incapacity and psychological and social damage to death [4] (…)"

**#15 -** L114: can you add the exact date (month, year) where data (ie; 11,000,000 new cases and 180,000 deaths worldwide every year, and in Brazil, there are around 1,000,000 incidents annually) were collected? Moreover add at least 2 references to argue your data

**Response:** Thank you for the comment. The worldwide data was collected from the World Health Organization Fact Sheet dated October 2023 (https://www.who.int/en/news-room/fact-sheets/detail/burns). We updated the Brazilian data according to the most recent data from the Brazilian Department of Health Epidemiological Bulletin. The changes are quoted below.

"(…) As per a World Health Organization Fact Sheet dated October 2023, there are over 11,000,000 cases worldwide annually, resulting in 180,000 deaths [5]. Only in Brazil, from 2015 to 2020, there were 19,772 deaths from burns, as delineated by data provided by the Brazilian Department of Health [6]. (…)"

**#16 -** L116: the expression "of these" is related to what worldwide data or Brazilian data?

**Response:** Thank you for the comment. The expression was related to Brazilian data. However, the sentence containing this expression was deleted in the revised manuscript since we updated the data referring to Brazil, once we update the data referring to deaths due to burns in Brazil.

**#17 -** L130: Change "II represents severity.."

**Response:** Thank you for the comment. We rewrote the sentence as quoted below.

"When present, IIs significantly impact patient outcomes, increasing fluid needs during resuscitation, pulmonary complications, and mortality [12-14], serving as a marker of severity and an independent risk factor for death [13,14], especially in patients with over 20% of body surface area burned [15,16] (…)".

**#18 -** L132-134: correct the English style, since the sentence is unclear "In addition…….condition [14]

**Response:** Thank you for the comment. We excluded this sentence in the revised manuscript.

**#19 -** L157: add a reference after life support

**Response:** Thank you for the comment. We excluded this sentence in the revised manuscript.

**#20 -** L160: add a reference after complications

**Response:** Thank you for the comment. We excluded this sentence in the revised manuscript.

**#21 -** L166-178: the subsection OBJECTIVES must reintegrate the Introduction section (must be the third paragraph). Here is a proposition.

The primary objective of this study will be to evaluate the incidence and mortality in burn patients due to IIs. The secondary objectives will be to assess the evidence regarding the association between IIs and respiratory complications (pneumonia, airway obstruction, acute respiratory failure, acute respiratory distress syndrome - ARDS), IMV needed and complications in other organ systems, and highlight factors associated with IIs in burn patients and prognostic factors associated with acute respiratory failure, IMV need and mortality in burn patients with IIs.

**Response:** Thank you for the comment and suggestion. We included the study objectives in the Introduction, as quoted below.

"In this context, the primary purpose of this study will be to assess the evidence regarding the frequency and mortality of II in burn patients. The secondary purposes will be to assess the evidence regarding the association between IIs and respiratory complications (pneumonia, airway obstruction, acute respiratory failure, acute respiratory distress syndrome - ARDS), need for IMV and complications in other organ systems, and highlight factors associated with IIs in burn patients and prognostic factors associated with acute respiratory failure, need for IMV and mortality of II in burn patients."

METHODS
**#22 -** MAJOR POINT: avoid redundancy between text and tables 1 and 2.

**Response:** Thank you for the comment. Some information that could cause redundancy between the text and the table was removed, but we kept Tables 1 and 2 to facilitate the visualization of the inclusion and exclusion criteria.

**#23 -** L185: delete (PROSPERO)

**Response:** Thank you for the comment. We rewrote the paragraph, as quoted below.

"This systematic literature review will be guided and reported according to the guidelines of the Preferred Reporting Items for Systematic Reviews and Meta-analysis (PRISMA) [23]. The protocol of this review is registered on the International prospective register of systematic reviews platform of the Center for Revisions and Disclosure of the University of York, United Kingdom (<https://www.crd.york.ac.uk/prospero>), under number RD42022343944".

**#24 -** L225: delete over 20 years of age

**Response:** Thank you for the comment. We rewrote the paragraph, as quoted below.

**"**Observational studies and clinical trials that evaluated the frequency and mortality of burn patients exposed to smoke inhalation.

".
**#25 -** L240: delete (RCTs)

**Response:** Thank you for the comment. We rewrote the paragraph, as quoted below.

"The search for studies will be performed without language and publication date restrictions in the following databases: (…)".

**#26 -** L254: MeSH not Mesh

L255-256: change as "Entree terms (https://www.embase.com)"

**Response:** Thank you for the comment. We rewrote the paragraph, as quoted below.

"In the search, descriptors previously identified in DeCS (Descriptors in Health Sciences, http://decs.bvs.br/), MeSH (Medical Subject Headings, https://www.nlm.nih.gov/mesh/meshhome.html), and Entree terms (https://www.embase.com) will be used, and their respective synonyms to include the largest number of relevant studies".

**#27 -** L282: over all the paper use the following model when describing the reviewers: (JA and DS in the authors' list).

**Response:** Thank you for the comment. We changed the reviewers' description through the text, as quoted below.

"Two reviewers will independently perform the studies' selection (JA and DS in the authors' list). The Rayyan platform provides an interface for each reviewer. Then, it indicates which studies showed disagreements in the analysis so that a third reviewer (AI in the authors' list) can resolve them.

Initially, the title and abstract will be analyzed. Next, the third reviewer (AI in the authors' list) will analyze the inclusion and exclusion disagreements about a particular study. Then, the texts will be fully evaluated, and the studies composing the review will be defined.

Again, for studies with a disagreement between the two main reviewers, the third reviewer (AI in the authors' list) will resolve the conflicts. Studies not meeting the inclusion criteria will be excluded, and the reasons for this decision will be recorded."

"For this evaluation, the Newcastle Ottawa Scale tool will be used for the nonrandomized studies [26] and the RoB 2 (2019) version for the randomized controlled trials [27]. Two reviewers will independently evaluate the risk of bias of the included studies (JA and DS in the authors' list). The third reviewer (AI in the authors' list) will resolve the disagreements."

"For this evaluation, the criteria of the Grading of Recommendations Assessment, Development and Evaluation (GRADE) working group will be used [28]. GRADE assesses the quality of the evidence based on the assessment of five domains: risk of bias, imprecision, inconsistency, indirectness, and publication bias [28]. Two reviewers will independently evaluate the quality of evidence of the included studies (JA and DS in the authors' list). The third reviewer (AI in the authors' list) will resolve the disagreements."

**#28 -** L295: add a reference after "Newcastle Ottawa Scale Tool".

**Response:** Thank you for the comment. We included a citation, as quoted below.

"The nonrandomized eligible studies will be included in the risk of bias assessment stage through the Newcastle Ottawa Scale Tool [26] and randomized controlled trials through the Cochrane Risk of Bias 2 (RoB 2) tool 2019 version [27]."

"For this evaluation, the Newcastle Ottawa Scale tool will be used for the nonrandomized studies [26] and the RoB 2 (2019) version for the randomized controlled trials [27]. (…) ".

**#29 -** L318: how you will contact the studies' authors?

**Response:** Thank you for the comment. If necessary, the studies' authors will be contacted via email, requesting additional data for analysis. The changes made in the Abstract and Methods are quoted below.

"(…) When numerical data are missing, the authors will be contacted via e-mail, requesting additional data for analysis".

**#30 -** L325: delete (SMD)

**Response:** Thank you for the comment. We rewrote the paragraph, as quoted below.

"(…) Since the outcomes of interest will be evaluated with different scales and units, standardized measurements will be used to calculate the effect sizes, standard mean deviation, and 95% confidence intervals (95% CI)."

DISCUSSION
**#31 -** L356: add a reference after severe condition

**Response:** Thank you for the comment. We included a citation, as quoted below.

"Respiratory impairment is still a major challenge in clinical practice and a promising area for research, needing further studies to understand and act on this potentially severe condition [44]. (…)".

REFERENCES
**#32 -** Ref 1 to 4: please cite the English titles.

**Response:** Thank you for the comment. We included the English titles for References 1 to 4.

TABLE 1.
**#33 -** Population: 19 years not 17 years, sex not gender,
Outcomes: Secondary: need for, "mechanical ventilation" by "invasive mechanical ventilation"

**Response:** Thank you for the comment. We changed the text in Table1, as quoted below.

"Adult patients of both sexes victims of acute burn regardless of magnitude or cause".

"Patients under 19 years".

" Secondary: respiratory complications, need for IMV, complications in other organ systems, factors associated with IIs, and factors associated with acute respiratory failure, need for IMV and mortality".

**#34 -** Type of study: should you report that only RCT will be included (better that Observational studies). In fact, there is a discordance between text and table 1 regarding this point (see L240)

**Response:** Thank you for the comment. As we do not intend to assess the effect of any particular intervention on primary outcome, we also included observational studies instead of only randomized clinical trials. This choice is predicated on our judgment that the inclusion of observational studies is important to evaluate our study outcomes. We changed the text to correct the discordance, as quoted below.

"The search for studies will be performed without language and publication date restrictions in the following databases: (…)".

"Observational studies and clinical trials that evaluated the frequency and mortality of burn patients exposed to smoke Inhalation".

Table 1 – Type of study - "Observational studies and clinical trials"

**#35 -** Type of study: do will exclude letters to editor and editorials

**Response:** Thank you very much for carefully analyzing the study that strengthened the manuscript. We will exclude editorials. Letters to the editor that do not report results from original research will also be excluded. The changes in these exclusion criteria are quoted below.

"Studies presenting incomplete data, reviews, case series, case reports, and editorials will be excluded. Letters to the editor that do not report results from original data will also be excluded."

**Reviewer #4**

Dear authors

I have read with a great interest the manuscript titled "Incidence and mortality in burn patients due to inhalation injury: a systematic review and meta-analysis protocol".
The topic is promising and addresses a significant gap in burn injury researches.
Overall, I find the study design to be well-structured and relevant to the field of burn injury research.

**Response:** Thank you very much for the positive comments.

However, there are a few key points that may require revision and improvement:
Major comments
**#1 -** Scope and Focus of the study

The authors have outlined their objectives and outcomes effectively, focusing on incidence and mortality related to inhalation injury in burn patients. However, I suggest that the scope of the study could be enhanced by including the clinical presentation and the severity of patients included in the analysis. Characterizing a distinct phenotype of patients at risk could provide valuable insights into the management and prognosis of inhalation injuries. By delving deeper into patient characteristics and clinical profiles, the study can potentially offer a more comprehensive understanding of this critical medical condition.

**Response:** Thank you for the comment. We included in the secondary purposes factors associated with acute respiratory failure, need for IMV, and mortality. The identification of these factors may contribute to defining the phenotype of II that is associated with IMV and poor prognosis.

The changes are quoted below.

INTRODUCTION

"In this context, the primary purpose of this study will be to assess the evidence regarding the frequency and mortality of II in burn patients. The secondary purposes will be to assess the evidence regarding the association between IIs and respiratory complications (pneumonia, airway obstruction, acute respiratory failure, acute respiratory distress syndrome - ARDS), need for IMV and complications in other organ systems, and highlight factors associated with IIs in burn patients and prognostic factors associated with acute respiratory failure, need for IMV and mortality of II in burn patients."

METHODS

"Outcomes evaluated

The primary outcomes will be:

- Frequency;
- Mortality.

Secondary outcomes will be:

- Respiratory complications: pneumonia, airway obstruction, acute respiratory failure, and ARDS;
- Need for IMV;
- Complications in other organ systems;
- Factors associated with IIs, need for IMV, complications in other organ systems, and mortality".

DISCUSSION

"(…) In this aspect, especially the identification of factors associated with acute respiratory failure, need for IMV, and mortality may contribute to defining the phenotype of inhalation burns that is associated with poor prognosis and clinical approaches that may have led to better outcomes, which can contribute to stricter monitoring of these patients and the institution of earlier clinical therapeutic approaches to improve outcomes for these patients."

**#2 -** Furthermore, given the absence of a consensus on the management of these patients, it is essential to include an evaluation of different management strategies and their impact on outcomes. This addition would enhance the clinical relevance and applicability of the study's findings.

**Response:** Thank you for the comment. We changed the inclusion criteria to include clinical trials and observational studies. This will also make it possible to evaluate different management strategies as factors to prevent IMV, complications, and poor outcomes.

The changes are quoted below.

METHODS

"Observational studies and clinical trials that evaluated the frequency and mortality of burn patients exposed to smoke Inhalation".

DISCUSSION

"(…) In this aspect, especially the identification of factors associated with acute respiratory failure, need for IMV, and mortality may contribute to defining the phenotype of inhalation burns that is associated with poor prognosis and clinical approaches that may have led to better outcomes, which can contribute to stricter monitoring of these patients and the institution of earlier clinical therapeutic approaches to improve outcomes for these patients.".

**#2 -** Inhalation injury and poor prognosis
The incrimination of inhalation injury in the prognosis of patients as complex as burns must be handled carefully by the authors, given that several confounding factors can lead to poor prognosis in those patients, such as shock, delirium, infections. In the protocol, it is crucial for the authors to outline their strategy for addressing these confounding factors. Exploring subgroups based on the severity of confounding factors, such as the extent of shock or presence of infections, can provide valuable insights into how these variables interact with inhalation injury in determining patient prognosis.

**Response:** Thank you for the comment. To minimize the risk of bias due to ARDS-induced and other respiratory complications that may be induced by skin burns without IIs and it is a frequent cause of need for IMV, we will only include studies that compared burn patients with and without II to evaluate the association between IIs and respiratory complications (pneumonia, airway obstruction, acute respiratory failure, acute respiratory distress syndrome), and need for IMV and complications in other organ systems, and the factors associated with IIs in burn patients. We also included the possibility of exploring subgroups based on the severity of confounding factors in the statistical analysis and emphasized this aspect in the Discussion.

The changes are quoted below.

INTRODUCTION

"In this context, the primary purpose of this study will be to assess the evidence regarding the frequency and mortality of II in burn patients. The secondary purposes will be to assess the evidence regarding the association between IIs and respiratory complications (pneumonia, airway obstruction, acute respiratory failure, acute respiratory distress syndrome - ARDS), need for IMV and complications in other organ systems, and highlight factors associated with IIs in burn patients and prognostic factors associated with acute respiratory failure, need for IMV and mortality of II in burn patients."

METHODS

"Secondary outcomes will be:

- Respiratory complications: pneumonia, airway obstruction, acute respiratory failure, and ARDS;
- Need for IMV;
- Complications in other organ systems;
- Factors associated with IIs, need for IMV, complications in other organ systems, and mortality".

"Summary of results and statistical analysis

(…) For further comparisons concerning the extent of burn injury size, shock, or presence of infections, subgroup analysis will be performed if feasible.".

DISCUSSION

"Although most patients exposed to smoke inhalation evolve well, the development of respiratory injury significantly worsens the outcome of these patients with a significant increase in mortality and complications, including long-term sequelae [17,34-36]. Indeed, pulmonary complications following burns and II cause or directly contribute to 77% deaths [37,38]. Among the pulmonary complications, ARDS may develop early or several days after the exposure [39]. Although ARDS may also occur in burns without II, the clinical symptoms tend to worsen following IIs. In II, ARDS usually starts earlier, progresses with greater severity, and requires IMV for longer [13]. Furthermore, sepsis and acute respiratory failure are frequent causes of morbidity and mortality in patients with exclusive thermal burns, which may be even more prevalent in patients with IIs [13]."

**#3 -** Definitions
In my opinion it is essential that the protocol provides comprehensive and explicit definitions for all the studied outcomes and the exploratory factors employed. Precise definitions are vital to ensure consistency in the assessment of outcomes across included studies. For instance, if "respiratory complications" is a key outcome, the protocol should specify which complications are considered, such as pneumonia, acute respiratory distress syndrome (ARDS), bronchitis, or other relevant conditions.

**Response:** Thank you for the comment. We made changes to clarify the definition of respiratory complications, as quoted below.

INTRODUCTION

"In this context, the primary purpose of this study will be to assess the evidence regarding the frequency and mortality of II in burn patients. The secondary purposes will be to assess the evidence regarding the association between IIs and respiratory complications (pneumonia, airway obstruction, acute respiratory failure, acute respiratory distress syndrome - ARDS), need for IMV and complications in other organ systems, and highlight factors associated with IIs in burn patients and prognostic factors associated with acute respiratory failure, need for IMV and mortality of II in burn patients."

METHODS

"Secondary outcomes will be:

- Respiratory complications: pneumonia, airway obstruction, acute respiratory failure, and ARDS;
- Need for IMV;
- Complications in other organ systems;
- Factors associated with IIs, need for IMV, complications in other organ systems, and mortality.

The ARDS Berlin definition will be used to diagnose the ARDS [25]."

**#4 -** Likewise, the protocol should explicitly outline the criteria and methods utilized for diagnosing inhalation injury. Specify whether clinical assessment, bronchoscopy, imaging, or other diagnostic tools will be considered as valid methods for diagnosing inhalation injury. Additionally, discuss any threshold values or criteria used to define the presence or severity of inhalation injury based on these diagnostic methods.

**Response:** Thank you for the comment. We made changes to clarify the inhalation injury definition in the methods, as quoted below.

"Inhalation injury will be defined as the damage inflicted to the respiratory tract or lung tissue from smoke, heat, and/or chemical irritants introduced into the airway during a burn event [24]. Although bronchoscopy may be performed to confirm the diagnosis of II and is considered the gold standard for this type of evaluation, the studies that applied only clinical criteria or used imaging or laboratory findings for II diagnosis will be included in the review [10,11]."

**#5 -** Excluded studies
The exclusion of studies that evaluated specific interventions in patient victims of burns exposed to inhalation injury, should be explained by the authors.

**Response:** Thank you for the comment. We changed the inclusion and exclusion criteria to include clinical trials and observational studies. This will also make it possible to evaluate specific interventions as factors to prevent IMV, complications, and poor outcomes.

The changes are quoted below.

INCLUSION CRITERIA

"Observational studies and clinical trials that evaluated the frequency and mortality of burn patients exposed to smoke inhalation will be included".

EXCLUSION CRITERIA

"Studies in patients under 19 years of age will be excluded.

Studies presenting incomplete data, reviews, case series, case reports, and editorials will be excluded. Letters to the editor that do not report results from original data will also be excluded".

**#6 -** English Language Quality:
The manuscript would benefit from improvement in the quality of English language usage. There are several instances of grammatical errors and awkward phrasing, throughout the document. Therefore, I recommend a thorough language edit to ensure that the manuscript is easy to read and understand.

**Response:** Thank you for the observation. The English style was revised throughout the manuscript.

Generals comments

**#7 -** The title formulation of the manuscript could benefit from enhancement. For instance, consider revising it to: "Incidence and Mortality of Inhalation Injuries in Burn Patients: A Systematic Review and Meta-Analysis protocol."

**Response:** Thank you for the comment. We changed the title to "Frequency, complications, and mortality of inhalation injury in burn patients: a systematic review and meta-analysis protocol"

**#8 -** There is a confusion in the manuscript between prevalence and incidence, although I think it is actually a study of frequency.

**Response:** Thank you for the comment. We changed "incidence" and "prevalence" to "frequency" throughout the text to avoid any inconsistency.

**#9 -** There is a confusion in Age of excluded patients.

**Response:** Thank you very much for carefully analyzing the study that strengthened the manuscript. In Table 1 – Exclusion Criteria, we changed "under 17 years of age" to "under 19 years of age", as quoted below.

"Patients under 19 years".

**Reviewer #5**

I would like to extend my sincere appreciation for the opportunity to review the manuscript titled "Incidence and mortality in burn patients due to inhalation injury: a systematic review and meta-analysis protocol." It is both an honor and a privilege to contribute to the evaluation of this important work, which addresses a critical topic in the field of critical care, burn care and inhalation injury.

The significance of understanding the incidence and mortality associated with inhalation injuries in burn patients cannot be overstated, as it holds substantial implications for clinical practice and patient outcomes.

I commend the authors for their initiative in undertaking this research, and I look forward to conducting a thorough evaluation of the manuscript.

Thank you once again for entrusting me with the peer review of this manuscript, and I eagerly anticipate the opportunity to provide constructive feedback and contribute to the advancement of this vital field of study.

**Response:** Thank you for the positive comment.

I have a few general comments regarding the manuscript as a whole:

**#1 -** I noticed the use of both "prevalence" and "incidence" separately in different sections. As this might be a source of confusion, I suggest to stick to using "the prevalence and incidence of".

**Response:** Thank you for the comment. We changed "incidence" and "prevalence" to "frequency" throughout the text throughout the text to avoid any inconsistency.

**#2 -** This manuscript has quite a number of grammatical mistakes and I sometimes had trouble following sentences. I think thorough copyediting is necessary.

**Response:** Thank you for the observation. The English style was revised throughout the manuscript.

I have a few additional reservations I found within the manuscript
In the objectives:
**#3 -** I find that the primary objective is clear and directly addresses a fundamental aspect. It's essential to assess the incidence and mortality associated with inhalation injuries in burn patients to understand the severity and impact of these injuries.
However, I suggest grouping and incorporating the secondary outcomes into a common comprehensive one: to identify the clinical trajectory of the most severe patients integrating the circumstances and severity of the burn, time to onset of the acute respiratory failure, the need for IMV, and the occurrence of extra respiratory end-organ failure.

**Response:** Thank you for the suggestion. We changed the objectives as quoted below.

"In this context, the primary purpose of this study will be to assess the evidence regarding the frequency and mortality of II in burn patients. The secondary purposes will be to assess the evidence regarding the association between IIs and respiratory complications (pneumonia, airway obstruction, acute respiratory failure, acute respiratory distress syndrome - ARDS), need for IMV and complications in other organ systems, and highlight factors associated with IIs in burn patients and prognostic factors associated with acute respiratory failure, need for IMV and mortality of II in burn patients."

**#4 -** To this same end, I wondered the reason for excluding patients having underwent specific interventions? I think it would have been more interesting to summarize in a direct manner the different interventions and their association to the trajectory and phenotype of the most severe patients.

**Response:** Thank you for the comment. We changed the inclusion and exclusion criteria to include clinical trials and observational studies. This will also make it possible to evaluate specific interventions as factors to prevent IMV, complications, and poor outcomes.

The changes are quoted below.

INCLUSION CRITERIA

"Observational studies and clinical trials that evaluated the frequency and mortality of burn patients exposed to smoke Inhalation will be included".

EXCLUSION CRITERIA

"Studies in patients under 19 years of age will be excluded.

Studies presenting incomplete data, reviews, case series, case reports, and editorials will be excluded. Letters to editor that do not report results from original data will also be excluded".

**#5 -** You should clearly define what is considered as "respiratory complications". And more than that, specifically differentiate in studies between primary injuries associated to smoke inhalation and secondary complications such as ARDS.

**Response:** Thank you for the comment. Respiratory complications were defined as pneumonia, airway obstruction, acute respiratory failure, acute respiratory distress syndrome, and need for IMV. To minimize the risk of bias due to ARDS-induced and other respiratory complications that may be induced by skin burns without IIs and it is a frequent cause of need for IMV, we will only include studies that compared burn patients with and without II to evaluate the association between IIs and respiratory complications (pneumonia, airway obstruction, acute respiratory failure, acute respiratory distress syndrome), and need for IMV and complications in other organ systems, and the factors associated with IIs in burn patients. We also included the possibility of exploring subgroups based on the severity of confounding factors in the statistical analysis and emphasized this aspect in the Discussion.

The changes are quoted below.

INTRODUCTION

"In this context, the primary purpose of this study will be to assess the evidence regarding the frequency and mortality of II in burn patients. The secondary purposes will be to assess the evidence regarding the association between IIs and respiratory complications (pneumonia, airway obstruction, acute respiratory failure, acute respiratory distress syndrome - ARDS), need for IMV and complications in other organ systems, and highlight factors associated with IIs in burn patients and prognostic factors associated with acute respiratory failure, need for IMV and mortality of II in burn patients."

METHODS

"Secondary outcomes will be:

- Respiratory complications: pneumonia, airway obstruction, acute respiratory failure, and ARDS;
- Need for IMV
- Complications in other organ systems;
- Factors associated with IIs, need for IMV, complications in other organ systems, and mortality".

DISCUSSION

"Although most patients exposed to smoke inhalation evolve well, the development of respiratory injury significantly worsens the outcome of these patients with a significant increase in mortality and complications, including long-term sequelae [17,34-36]. Indeed, pulmonary complications following burns and II cause or directly contribute to 77% deaths [37,38]. Among the pulmonary complications, ARDS may develop early or several days after the exposure [39]. Although ARDS may also occur in burns without II, the clinical symptoms tend to worsen following IIs. In II, ARDS usually starts earlier, progresses with greater severity, and requires IMV for longer [13]. Furthermore, sepsis and acute respiratory failure are frequent causes of morbidity and mortality in patients with exclusive thermal burns, which may be even more prevalent in patients with IIs [13]."

**#6 -** In the methods section:
I noticed a discrepancy between the exclusion criteria in table I and those in the methods section (excluding those under 17 years of age in the table versus excluding those under 19). I would like to ask the authors to clarify this as I might have misunderstood the explanation behind it.

**Response:** Thank you for the comment. In Table 1 – Exclusion Criteria, we changed "under 17 years of age" to "under 19 years of age", as quoted below.

"Patients under 19 years".

**#7 -** I find that this protocol is able to provide important information in the management of such patients, if a few modifications are made in the methodology with the comprehensive originality of providing the clinical trajectory and phenotype of the most severe patients, those with the highest mortality rates. This would allow the final study to identify treatable traits in this modern era of precision therapy and precision medicine.

**Response:** Thank you very much for the positive comment and carefully analyzing the study that strengthened the manuscript.
